# Air travel during pregnancy and the risk of adverse pregnancy outcomes as gestational age and weight at birth: A retrospective study among 284,069 women in Israel between the years 2000 to 2016

**DOI:** 10.1371/journal.pone.0228639

**Published:** 2020-02-06

**Authors:** Hila Shalev Ram, Shai Ram, Netanella Miller, Yael Shalev Rosental, Gabriel Chodick

**Affiliations:** 1 Faculty of Medicine, Technion, Haifa, Israel; 2 Sackler Faculty of Medicine, Tel Aviv University, Tel Aviv, Israel; 3 Meir Medical Center, Kfar Saba, Israel; 4 Maccabi Healthcare Services, Tel Aviv, Israel Meir Medical Center, Kfar Saba, Israel; Ospedale dei Bambini Vittore Buzzi, ITALY

## Abstract

**Objective:**

The American College of Gynecology (ACOG) recommendation does not limit air travel during pregnancy, yet the evidence for air travel effect on adverse pregnancy outcomes is limited and debatable. Study objectives were to examine the association between air travel during pregnancy and preterm birth together with decreased birth weight.

**Study design:**

A retrospective cohort study.

**Methods:**

The study evaluated 628,292 women who gave birth to singleton infants from 9/2000 to 9/2016 and classified them into “air travel during pregnancy” or not, based on flight insurance as proxy. Multiple linear regression models were utilized to examine the relationship between air travel during pregnancy and newborn's gestational age and birth weight, while accounting for socioeconomic status, diabetes, high-risk pregnancies, and smoking.

**Results:**

A total of 41,677 (6.6%) births of women who air traveled during pregnancy was included, and 586,615 (93.4%) births of women who did not. Air travel during pregnancy was associated with a statistically significant (p<0.0001) but negligible increase in birth weight (9 gr. 95% CI: 4.8 to 14.5 gr.) and gestational age (0.36 days. 95% CI: 0.24–0.48).

**Conclusion:**

The study results provide no evidence that air travel during pregnancy is related to adverse effects on gestational age or birth weight. These findings corroborate the current recommendations of ACOG.

## Introduction

Worldwide, air travel has grown steadily over the years with the number of travelers mounting from one billion in 1990 to four billion in 2017[1]. Due to this increase, it is necessary to relate to potential health hazards associated with air travel, including adverse birth outcomes in pregnant women.

The notion that air travel may affect the course of pregnancy is based on environmental changes during the flight. In an average commercial flight at about 35,000 ft, the cabin pressure decreases from atmospheric pressure of 760 mmHg to about 560 mmHg. Consequently, oxygen saturation drops, during both long and short flights in all age groups, to a mean saturation of 90–93% in a healthy population and as low as 80% in patients with cardiopulmonary diseases[2,3]. Fibrinolytic activity is reduced by the relative hypoxia in the cabin and leads to a release of vein wall relaxing factors[4], that might enhance venous stasis. Moreover, even in normal pregnancies, uterine artery blood flow, which can help compensate for the altered blood flow at altitude, was found to be reduced during flights[5]. Therefore, the effect of cabin pressurization, especially in complicated pregnancies with compromised uteroplacental blood flow is concerning.

Despite the plausible impact of environmental and physiological changes during a flight on pregnancy, and consequently on adverse pregnancy outcomes such as preterm delivery or low birth weight, data regarding this issue are scarce and controversial. A few studies found a significantly increased risk of preterm birth [6,7] and lower weight at birth[6] among women who have air traveled during pregnancy, while other studies have suggested no effect[8–10]. However, these studies, were limited in size, restricted to populations with predisposing factors, or lacked an adjustment for important confounding factors.

Despite the inconclusive literature, the American College of Obstetricians and Gynecologists’ (ACOG) stated in their last update, that in the absence of obstetric or medical complications, air travel is safe [11]. This recommendation is not supported by high-level evidence. The present analysis was conducted in order to fill the gap of direct, well-powered studies on the association between flying during pregnancy and adverse birth outcomes, namely low gestational age at birth and low birth weight.

## Materials & methods

### Study population

This study was conducted using the computerized data of Maccabi healthcare services, a 2.3 million patient integrated care organization in Israel, which covers 22–25% of the pregnant population in Israel[12]. The study protocol has been reviewed and approved by the Bait Balev Medical Center Institutional Review Board with document number 0026-18-BBL. Ethics committee waived the requirement for informed consent as this was a retrospective study. All data was fully anonymized before analysis.

Maccabi healthcare services’ female members, who gave birth during the study observation period—2000 through 2016, were eligible for analysis. Stillbirths, births under gestational age of 24 weeks and women who had multiple gestations were excluded, leaving 717,551 women who met eligibility criteria. Out of those, 89,259 (12.4%) were excluded due to, missing data on gestational age or birth weight, leaving 628,292 observations in the final cohort. Approximately 97% of the 20.1 million air travels per year in Israel are international[13]. The travel insurance rates in Israel are about 95% according to the Israel consumers council[14] as in other well-developed countries, such as Australia, in which the travel insurance rates are 92%[15]. Furthermore, domestic air travels in Israel, which do not require health insurance, are relatively short (up to 45 min). The cooperation between “Maccabi healthcare services” with “Clal Insurance Enterprises Holdings Ltd”, one of the largest insurance companies in Israel (which provides discounted flight insurance for Maccabi healthcare members), assisted in accessing women who air traveled by air. Data regarding insurance policy purchases among the study population have been collected. In order to detect if a woman traveled by air during pregnancy, each policy day of inbound and outbound flights were obtained.

### Study outcomes and other study variables

Adverse pregnancy outcomes, including low birth weight and low gestational age at birth, were the primary outcomes of interest. These variables were analyzed as continuous variables and also as binary variables (for low birth weight under 2500 grams and for gestational age under 37 weeks). Data regarding date of birth, gestational week at birth and birth-weight were extracted from the Maccabi healthcare services database since this information is routinely provided to Maccabi healthcare services by the Israeli Ministry of Health.

*“*High-risk” pregnancies were identified through “Maccabi healthcare services” computer database of women diagnosed as "high-risk” pregnancy. Women with “high-risk" pregnancies are diagnosed in ‘Maccabi Healthcare Services’ computerized database if the pregnancy threatens the health or life of the mother or her fetus. This includes women with e**xisting health conditions**, such as high blood pressure, diabetes, kidney diseases; or a history of recurrent pregnancy loss[16], preterm birth, or other problems with previous pregnancy. Pregnancies with medical conditions that occur during pregnancy such as intrauterine growth restriction of the fetus, recurrent bleeding, placenta previa and gestational diabetes of pregnancy are also diagnosed as “high-risk” pregnancies. Other collected data included the number of siblings, gestational age at delivery, diagnosis of pre-gestational diabetes mellitus, gestational diabetes mellitus (GDM) and pre-eclampsia. Pre-eclampsia, gestational diabetes, and pre-gestational diabetes were taken from electronic health records and were diagnosed by the assignment of the International Classification of Diseases, 9th Revision, Clinical Modification (ICD-9-CM) codes. Also, data regarding fertility treatments, including in-vitro fertilization (IVF) and non-IVF fertility treatments, were obtained. Smoking status and socio-economic status were derived directly from the electronic health records for the participants. The level of socioeconomic status (SES) was assessed according to the poverty index of the child's address enumeration area, as defined by the 2008 national census [17]. The poverty index is based on several parameters, including household income, educational qualifications, crowding, material conditions, and car ownership, census of population, and housing. SES levels range between 1 (lowest) to 10 (highest). In this study, SES below four was considered as low SES, SES between 4 to 7 was considered medium SES, and SES between 8 to 10 was considered as high SES.

### Statistical analysis

Descriptive statistics were used to assess the distribution of variables; continuous variables were summarized as mean values with standard deviations, and categorical variables were summarized as counts and percentages. To ensure that pregnancy outcomes during the study period were most likely not constituted with an external cause, the multivariant analysis included known risk factors that affect preterm labor and weight at birth such as age, high-risk pregnancies, fertility treatments, smoking, socioeconomic status, number of siblings, and pre-eclampsia. Several multivariable analyses with birth weight in grams and gestational age at birth in days, as continuous variables were performed by multiple linear regression modeling, estimated by using ordinary least squares. Low birth weight (under 2,500 gram) and preterm birth (under 37 weeks), as binary dependent variables, were also examined and analyzed by logistic regression **(presented in Tables G and H in S1 Appendix)**.

Other measurements of exposure were examined, including the effect of the number of flights and timing of flights by trimesters during the index pregnancy **(presented in Tables D-F in S1 Appendix)**. “Exposure time” is the potential number of weeks that a pregnant woman might undertake air travel. Women who have experienced preterm birth, had shorter exposure times, which could have had an impact on the results. Thus, we conducted another analysis (**presented in Tables B and C in S1 Appendix),** which was limited to women who air-traveled before a gestational age of 32 weeks and gave birth after 32 weeks, meaning that each woman had a 32 week “exposure time”. The same analysis was done for 34 weeks as well.

The threshold for statistical significance was *P* < 0.05. Statistical analyses were performed using IBM-SPSS' GLM (general linear model) procedure (IBM Corp. Released 2017. IBM SPSS Statistics for Windows, Version 25.0. Armonk, NY).

## Results

A total of 628,292 live births was included in the study. Among the study population, 6.6% air traveled during pregnancy. Compared to unexposed pregnancies, women who did air travel during pregnancy were older (31.8±4.8years vs. 30.1±5.4 years) and of higher SES level (7.1±1.7 vs. 5.8 ±2.0). Furthermore, women who air traveled had higher prevalence of high-risk pregnancies (10.5% vs. 7.6%) gestational diabetes mellitus (GDM) (7.7% vs. 6.3%) and fertility treatments (including IVF) (8.8% vs. 5.8%) than the unexposed group, but lower rates of smoking during pregnancy (4.4% vs. 5.9%) (p<0.0001 for these variables, **Table 1)**.

**Table 1:** Characteristics of study populations (N = 628,292).

	Air travel- No	Air travel- Yes	
	N = 586,615 (93.4%)	N = 41,677 (6.6%)	
Variable	Mean (±SD)	Mean (±SD)	P-value
Mother's age	30.1 (±5.4)	31.8 (±4.8)	<0.0001
BMI before pregnancy	25.7 (±5.3)	24.7 (±1.8)	<0.0001
Number of Siblings	1.9 (±1.5)	1.6 (±1.19)	<0.0001
Socioeconomic state	5.8 (±2.0)	7.1 (±1.7)	<0.0001
	**N (%)**	**N (%)**	**P-value**
Gestational DM	36677 (6.3%)	3255 (7.7%)	<0.0001
Pre-eclampsia	10972 (1.9%)	854 (2%)	0.10
IVF	18007 (3.1%)	1853 (4.4%)	<0.0001
Other fertility treatments	34152 (5.8%)	3747 (8.8%)	<0.0001
High risk pregnancy	44503 (7.6%)	4457 (10.5%)	<0.0001
Smoking during pregnancy	34494 (5.9%)	1866 (4.4%)	<0.0001

**Fig 1:**
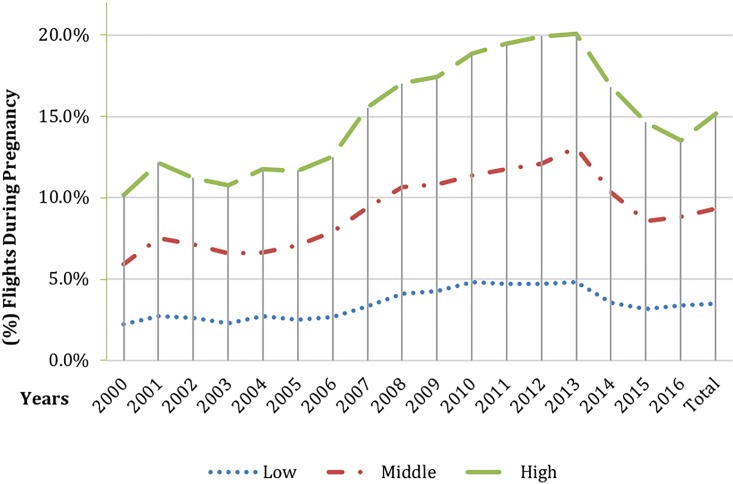
Flights during pregnancy according to low (SES 1–5), medium (SES 6–7) and high (SES 8–10) socioeconomic status between the years 2000 to 2016.

**Fig 2:**
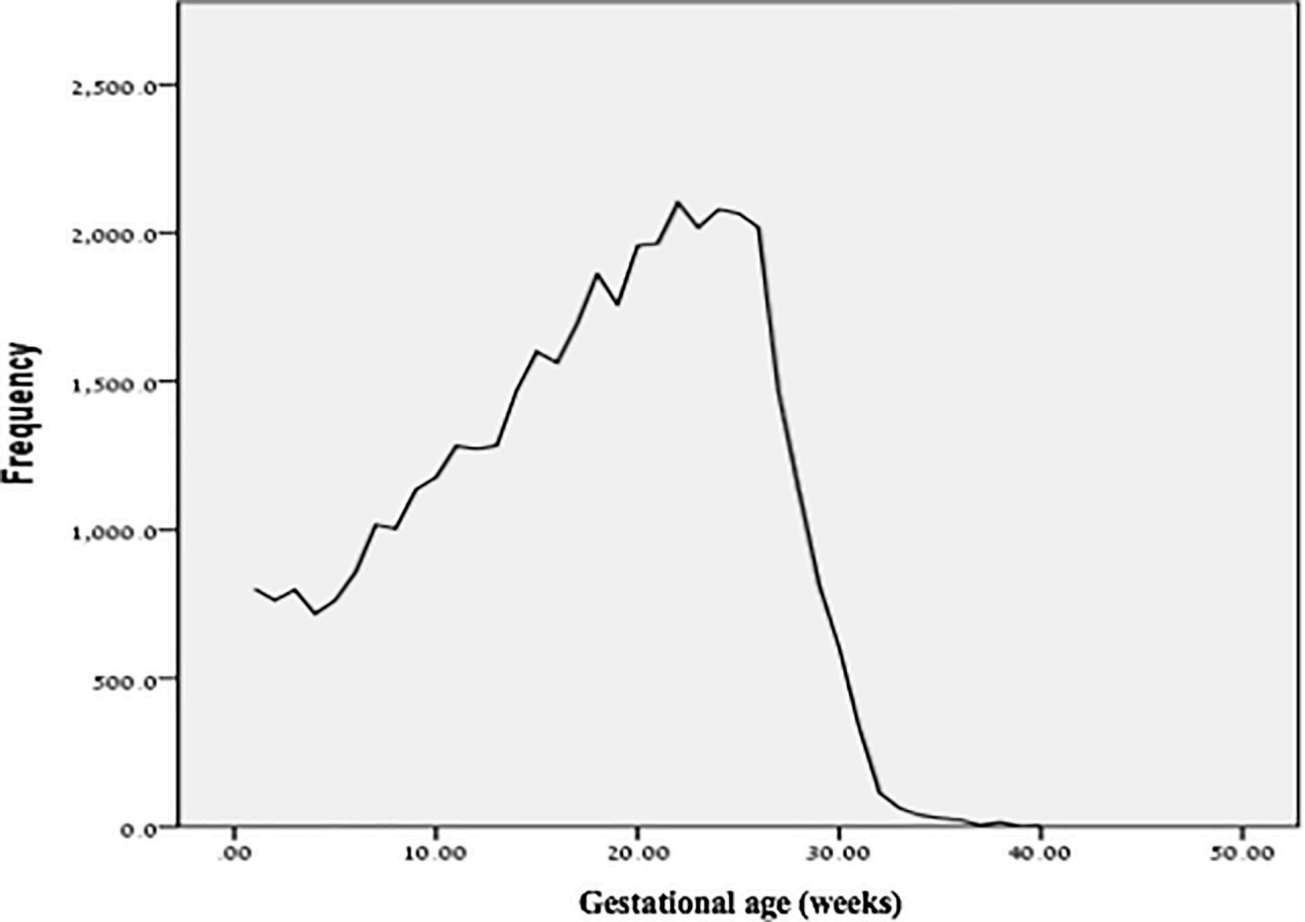
Frequency of air travel during pregnancy by gestational age.

During the study period, the proportion of women who air traveled during pregnancy has increased between the years 2000 and 2014, particularly among higher SES groups (**Fig 1)**. As presented in **Fig 2**, the majority of women that air traveled during the second trimester, with a growing proportion traveling as late as mid-third trimester. Air travel during pregnancy was associated with neglectable effects on gestational age (39+0 ±1.6 weeks in air travel group vs 39+1 ±1.7 weeks in control group, p-value <0.0001) and weight at birth **(**3263 ±477 grams in air travel group vs 3269±492 grams in control group, p-value = 0.01) (**see Table A in S1 Appendix**).

In a multivariable model for weight at birth, air travel during pregnancy was associated with a statistically significant yet clinically neglectable increase of 9 grams (95% CI:4.8–14.5, p-value <0.0001). Maternal gestational diabetes mellitus and siblings were associated with increased birth weight as well, while a negative association was calculated for high-risk pregnancies, pre-eclampsia, smoking, and fertility treatments (**Table 2**). Similarly, in the multivariable model for gestational age at birth, air travel during pregnancy was associated with a minimal increase of 0.36 days (95%CI:0.24–0.48, p-value <0.0001). The most significant association was calculated for high-risk pregnancies, with a decrease of more than eight days. All other variables decreased the gestational age differently (**Table 3**).

**Table 2:** Multiple linear regression model for birth weight.

		95% Confidence Interval		
Variables	B	Lower	Upper	
	(grams)			P-value
Mother's age	2.2	2.0	2.5	<0.0001
Number of Siblings	25.2	24.4	26.1	<0.0001
Gestational DM	74.1	69.1	79.1	<0.0001
Pre-eclampsia	-160.2	-169.1	-151.4	<0.0001
IVF	-76.0	-83.6	-68.3	<0.0001
Other fertility treatments	-84.8	-90.4	-79.2	<0.0001
High risk Pregnancy	-223.8	-228.3	-219.2	<0.0001
Smoking	-54.0	-59.1	-48.8	<0.0001
Socioeconomic state	-3.1	-3.7	-2.4	<0.0001
Air travel during pregnancy	9.7	4.8	14.5	<0.0001

**Table 3:** Multiple linear regression model for gestational age at birth.

		95% Confidence Interval		
Variables	B	Lower	Upper	P value
	(Days)			
Mother's age	-0.13	-0.14	-0.13	<0.0001
Number of Siblings	0.25	0.22	0.27	<0.0001
Gestational DM	-2.04	-2.16	-1.93	<0.0001
Preeclampsia	-5.33	-5.54	-5.12	<0.0001
IVF	-2.79	-2.98	-2.60	<0.0001
Other fertility treatments	-1.17	-1.30	-1.04	<0.0001
High risk Pregnancy	-8.79	-8.90	-8.68	<0.0001
Smoking	-1.34	-1.46	-1.22	<0.0001
Socioeconomic state	-0.24	-0.25	-0.22	<0.0001
Air travel during pregnancy	0.36	0.24	0.48	<0.0001

Additionally, frequency of air travels per pregnancy, timing of the flight during pregnancy (first, second or third trimester), analysis for preterm birth (under 37 weeks) and low birth weight (under 2,500 gram) as dependent categorial variables, and analysis of which we removed the effect of "exposure time" on pregnancy outcomes were examined by multivariable models. All analyses have shown similar results (see **Tables B-E in S1 Appendix**).

## Comment

### Principal findings of the study

In this observational retrospective cohort study of over 628 thousand live births, a statistically significant but clinically neglectable difference in birth weight and gestational age was found between women who air traveled during pregnancy and women who did not. The clinically non-significant results were consistent across specified subgroups and different timing of exposure. Moreover, the study showed a rise in air travels during pregnancy and a correlation to socio-demographical status. Women of a high SES flew more during pregnancy than women of a lower SES. Also, the data reveals that most flights occur during the second trimester.

### Results of the study in the context of other observations

This large cohort study supports the findings of a smaller study on 222 singleton pregnancies women[8], which did not find a correlation between air travel during pregnancy and preterm birth. However, except for a small cohort, this study did not take into account important confounders such as smoking, GDM, fertility treatments, and socioeconomic differences. Another retrospective cohort study which compared pregnancy outcomes between 3,693 flight attendants to all other births in Norway [9], found that the risk of low birth weight was lower for the female cabin attendants than for the referents, and suggested no increased risk of preterm birth. This study did not consider important confounders as well.

However, a study conducted by Chibber et al. on 992 pregnant women [6], found that air travel was significantly associated with increased risk of preterm birth between 34 and 37 weeks (adjusted odds ratio 1.5, 95% CI: 1.2, 1.8). The dramatic differences between exposed and unexposed women in birth-weights (2684 ± 481 g vs. 3481 ± 703 g) as well as in gestational age at birth (36.1 ± 0.8 vs. 39.2 ± 2.1) and significant racial differences raise concerns regarding residual confounders which were not considered. Our finding of which most women fly during the second trimester could be explained by ACOG’s official recommendation, which claims that the most common obstetric emergencies occur in the first and third trimesters[11] and also by the limitations of insurance companies for pregnant women[18].

### Strengths and limitations

The main strengths of this study include: (1) the sample size, which is, to the best of our knowledge, the largest ever used, (2) the accurate information obtained on many risk factors that may affect gestational age and birth weight, which provided the ability to conduct appropriate statistical analysis, (3) the consistency of the results when analyses were stratified for gestational age at birth, frequency of air travel, or timing of the flight during pregnancy, (4) some of this study’s results reinforce its reliability, by demonstrating well known negative effects on gestational age and birth weight of high-risk pregnancies, pre-eclampsia, smoking during pregnancy and fertility treatments and positive effect on weight at birth in women with GDM [19–23].

Although this study was conducted on a large and diverse database, unfortunately not all the essential information was available. For example, information about destinations and flight length. However, according to the Ben Gurion annual report[13], the vast majority (83%) of the flights taken, was to destinations with flight duration of between 2 to 6 hours, while 4% were under 2 hours and the rest (13%) are over 6 hours [24]. Thus, we can carefully assume that our dataset includes a range of about 6 hours for over 80% of the flights. We believe, that if there was an effect of short air travel (up to 6 hours), assuming they are the vast majority of flights during pregnancy, on gestational age or weight of birth, we would have seen a difference between the groups in the multiple analysis. Yet, we cannot claim based on this data, that air travel is safe for a long duration of flights (More than 6 hours). Another limitation is that domestic air travel in Israel, which is relatively short (up to 45 min) and does not require health insurance, was classified as non-travelers supposing they didn’t fly international. Moreover, it can be assumed that some women purchased their flight health insurance via other insurance companies rather than ‘Clal’ or did not buy flying insurance at all. However, members of ‘Maccabi’ get a discounted flight insurance through ‘Clal’ and therefore it is likely that a significant portion of women bought their insurance through ‘Clal’. We assume that the proportion of women that did not buy insurance at all or bought an insurance through ‘Clal’ and did not fly is negligible in such big cohorts. Another limitation of our study is that women with high-risk pregnancies who flew during a pregnancy had fetuses of a higher birth weight which may result in self-selection bias.

### Implications for research

Given the raise of pregnant women air travelling, and the various factors that may adversely affect birth outcomes during flights (cabin pressurization[2,3] and maternal physiological changes[25,26], other pregnancy adverse events such as pregnancy loss, thromboembolism, and newborn's health should be investigated in similarly large and unselected cohorts. Moreover, the effect of long air travel (more than 6 hours) should be thoroughly investigated.

### Conclusion

To conclude, the statistically significant difference in birth weight and gestational age between women who air traveled during pregnancy and women who did not was clinically neglectable. These small effects of flights on gestational age and birthweight are reassuring for pregnant women and their clinicians to fly during pregnancy, especially for short flights, and provide concrete statistical evidence in line with and corroborating the current recommendations of ACOG. Data are needed for a wider range of flight durations, including long flights.

## Supporting information

S1 Appendix Tables A-H. (DOCX)
