# Supplementary material for: Air travel during pregnancy and the risk of adverse pregnancy outcomes as gestational age and weight at birth: A retrospective study among 284,069 women in Israel between the years 2000 to 2016
Source: PLoS One. 2020 Feb 6;15(2):e0228639. doi: 10.1371/journal.pone.0228639 (PMC7004371; doi:10.1371/journal.pone.0228639)
Supplement: S1 Appendix — (DOCX) [file pone.0228639.s001.docx]

# **Appendix**

**Table A.** **Means of Weight and Gestational Age at Birth by Air travel And Control group.**

|  | **Air travel- No** | **Air travel- Yes** |  |
| --- | --- | --- | --- |
| **Variable** | **Mean (±SD)** | **Mean (±SD)** | **P value** |
| Gestational Age | 39+1 (±1.7) | 39+0 (±1.6) | <0.0001 |
| Weight at Birth | 3269 (±492) | 3263 (±477) | 0.011 |

**Tables B and C.** **Multiple Linear Regression Models for Birth Weight of Fetus, Without the Confounding Effect of “Exposure Time” To Flight.**

**Table B. Multiple Linear Regression Model for women who air traveled until gestational age of 32 and gave birth after gestational age of 32.**

| **Variables** | **B** | **95% Confidence Interval** | |  |
| --- | --- | --- | --- | --- |
|  | **(Grams)** | **Lower** | **Upper** | **P value** |
| Mother's age | 1.286 | 0.372 | 2.201 | 0.006 |
| Siblings | 35.467 | 31.889 | 39.044 | <0.0001 |
| Gestational DM | 48.973 | 33.854 | 64.091 | <0.0001 |
| Pre-eclampsia | -170.867 | -198.72 | -143.015 | <0.0001 |
| IVF | -41.408 | -63.448 | -19.369 | <0.0001 |
| Other fertility treatments | -80.593 | -96.36 | -64.826 | <0.0001 |
| High risk pregnancy | -148.46 | -161.557 | -135.363 | <0.0001 |
| Smoking | -31.976 | -51.244 | -12.708 | 0.001 |
| Socioeconomic state | -5.068 | -7.504 | -2.632 | <0.0001 |
| Air travel during pregnancy | 30.822 | 20.253 | 41.391 | <0.0001 |

**Table C. Multiple Linear Regression Model for women who air traveled until gestational age of 34 and gave birth after gestational age of 34.**

| **Variables** | **B** | **95% Confidence Interval** | |  |
| --- | --- | --- | --- | --- |
|  | **(Grams)** | **Lower** | **Upper** | **P value** |
| Mother's age | 1.304 | 0.41 | 2.198 | 0.004 |
| Siblings | 34.601 | 31.11 | 38.093 | <0.0001 |
| Gestational DM | 51.67 | 36.849 | 66.491 | <0.0001 |
| Pre-eclampsia | -139.068 | -166.624 | -111.512 | <0.0001 |
| IVF | -34.6 | -56.24 | -12.96 | 0.002 |
| Other fertility treatments | -77.004 | -92.467 | -61.541 | <0.0001 |
| High risk pregnancy | -130.427 | -143.315 | -117.538 | <0.0001 |
| Smoking | -29.707 | -48.568 | -10.845 | 0.002 |
| Socioeconomic state | -5.146 | -7.523 | -2.769 | <0.0001 |
| Air travel during pregnancy | 26.359 | 15.999 | 36.719 | <0.0001 |
|  | | | | |
| **Table D.** **Multiple Linear Regression Model for Birth Weight of Fetus, Including Number of Flights During Pregnancy.**   \| **Variables** \| \| **B (grams)** \| **95% Wald Confidence Interval** \| \|  \| \| --- \| --- \| --- \| --- \| --- \| --- \| \| **Lower** \| **Upper** \| **P value** \| \| Maternal age \| \| 2.474 \| 2.232 \| 2.716 \| <0.0001 \| \| Gestational DM \| \| 63.27 \| 58.48 \| 68.07 \| <0.0001 \| \| Pre-eclampsia \| \| -166.91 \| -175.39 \| -158.43 \| <0.0001 \| \| IVF \| \| -57.47 \| -63.99 \| -50.94 \| <0.0001 \| \| Other fertility treatments \| \| -79.74 \| -84.82 \| -74.67 \| <0.0001 \| \| High risk \| \| -146.36 \| -153.38 \| -139.35 \| <0.0001 \| \| Smoking \| \| 39.04 \| 26.61 \| 51.47 \| <0.0001 \| \| Socioeconomic state \| \| -12.17 \| -15.66 \| -8.67 \| <0.0001 \| \| Sequential count of matching cases \| \| 33.29 \| 32.38 \| 34.19 \| <0.0001 \| \| Number of flights during pregnancy (N) \| 7+ (325) \| -33.678 \| -86.361 \| 19.004 \| 0.21 \| \|  \| 6 (911) \| 7.024 \| -24.472 \| 38.52 \| 0.662 \| \|  \| 5 (89) \| -83.405 \| -184.033 \| 17.222 \| 0.104 \| \|  \| 4 (5,620) \| 0.551 \| -12.211 \| 13.313 \| 0.933 \| \|  \| 3 (379) \| -1.333 \| -50.114 \| 47.448 \| 0.957 \| \|  \| 2 (42,990) \| 1.287 \| -3.509 \| 6.083 \| 0.599 \| \|  \| 1 (991) \| 8.852 \| -21.331 \| 39.035 \| 0.565 \| | | | | |

**Table E.** **Multiple linear regression model for weight of fetus at birth, including the timing of air traveling during pregnancy by first, second, and third trimesters.**

| **Variables** | | **B** | **95% Confidence Interval** | |  |
| --- | --- | --- | --- | --- | --- |
|  | | **(Grams)** | **Lower** | **Upper** | **P value** |
| Mother's age | | 2.208 | 1.961 | 2.456 | <0.0001 |
| Sibling | | 25.238 | 24.349 | 26.127 | <0.0001 |
| Gestational DM | | 74.101 | 69.122 | 79.081 | <0.0001 |
| Preeclampsia | | -160.247 | -169.093 | -151.401 | <0.0001 |
| IVF | | -75.96 | -83.644 | -68.276 | <0.0001 |
| Other fertility treatments | | -84.802 | -90.418 | -79.187 | <0.0001 |
| High risk Pregnancy | | -223.779 | -228.32 | -219.238 | <0.0001 |
| Smoking | | -53.971 | -59.109 | -48.833 | <0.0001 |
| Socioeconomic state |  | -3.063 | -3.712 | -2.414 | <0.0001 |
| Air travel during pregnancy | First trimester | 9.869 | 2.349 | 17.389 | 0.01 |
|  | Second trimester | 6.664 | 0.475 | 12.853 | 0.035 |
|  | Third trimester | 22.115 | 5.167 | 39.064 | 0.011 |

**Table F. Multiple linear regression model for gestational age at birth, including the timing of air traveling during pregnancy by first, second, and third trimesters.**

| **Variables** | | **B** | **95% Confidence Interval** | |  |
| --- | --- | --- | --- | --- | --- |
|  | | **(Days)** | **Lower** | **Upper** | P value |
| Mother's age | | -0.133 | -0.14 | -0.126 | <0.0001 |
| Siblings | | 0.245 | 0.224 | 0.266 | <0.0001 |
| Gestational DM | | -2.044 | -2.163 | -1.918 | <0.0001 |
| Preeclampsia | | -5.327 | -5.537 | -5.11 | <0.0001 |
| IVF | | -2.793 | -2.975 | -2.604 | <0.0001 |
| Other fertility treatments | | -1.169 | -1.302 | -1.036 | <0.0001 |
| High risk Pregnancy | | -8.792 | -8.897 | -8.68 | <0.0001 |
| Smoking | | -1.337 | -1.463 | -1.218 | <0.0001 |
| Socioeconomic state |  | -0.238 | -0.252 | -0.224 | <0.0001 |
| Air travel during pregnancy | First trimester | -0.042 | -0.224 | 0.14 | 0.658 |
|  | Second trimester | 0.427 | 0.273 | 0.574 | <0.0001 |
|  | Third trimester | 1.806 | 1.4 | 2.219 | <0.0001 |

**Table G.** **Logistic regression analysis for preterm birth (under 37 weeks) as a dependent categorial variable.**

| **Variables** | **Odd Ratio** | **95% Confidence Interval** | |  |
| --- | --- | --- | --- | --- |
|  |  | **Lower** | **Upper** | **P-value** |
| Mother's age | 1.014 | 1.012 | 1.017 | <0.0001 |
| Siblings | 0.935 | 0.927 | 0.943 | <0.0001 |
| Gestational DM | 0.976 | 0.936 | 1.017 | 0.249 |
| Preeclampsia | 2.588 | 2.455 | 2.728 | <0.0001 |
| IVF | 1.563 | 1.481 | 1.651 | <0.0001 |
| Other fertility treatments | 1.232 | 1.178 | 1.289 | <0.0001 |
| High risk Pregnancy | 4.133 | 4.02 | 4.249 | <0.0001 |
| Smoking | 1.268 | 1.214 | 1.324 | <0.0001 |
| Socioeconomic state | 0.99 | 0.984 | 0.996 | <0.0001 |
| Air travel during pregnancy | 0.868 | 0.829 | 0.909 | <0.0001 |

**Table H.** **Logistic regression analysis for low birth weight (under 2,500 gram) as a dependent categorial variable.**

| **Variables** | **Odd Ratio** | **95% Confidence Interval** | |  |
| --- | --- | --- | --- | --- |
|  |  | **Lower** | **Upper** | **P-value** |
| Mother's age | 1.007 | 1.005 | 1.009 | <0.0001 |
| Siblings | 0.878 | 0.87 | 0.886 | <0.0001 |
| Gestational DM | 0.779 | 0.744 | 0.815 | <0.0001 |
| Preeclampsia | 2.6 | 2.467 | 2.74 | <0.0001 |
| IVF | 1.386 | 1.313 | 1.464 | <0.0001 |
| Other fertility treatments | 1.486 | 1.424 | 1.551 | <0.0001 |
| High risk Pregnancy | 3.416 | 3.32 | 3.514 | <0.0001 |
| Smoking | 1.408 | 1.352 | 1.467 | <0.0001 |
| Socioeconomic state | 0.974 | 0.968 | 0.98 | <0.0001 |
| Air travel during pregnancy | 0.889 | 0.849 | 0.931 | <0.0001 |
